# Annexin A1/Formyl Peptide Receptor Pathway Controls Uterine Receptivity to the Blastocyst

**DOI:** 10.3390/cells9051188

**Published:** 2020-05-11

**Authors:** Cristina B. Hebeda, Silvana Sandri, Cláudia M. Benis, Marina de Paula-Silva, Rodrigo A. Loiola, Chris Reutelingsperger, Mauro Perretti, Sandra H. P. Farsky

**Affiliations:** 1 Department of Clinical and Toxicological Analyses, School of Pharmaceutical Sciences, University of Sao Paulo, São Paulo CEP 05508-000, Brazil; crisbh@gmail.com (C.B.H.); ssandris@gmail.com (S.S.); claudiabenis@usp.br (C.M.B.); mpsilva.bio@gmail.com (M.d.P.-S.); rodrigoazl@gmail.com (R.A.L.); 2 Faculty of Health, Medicine and Life Sciences, Part of Maastricht University Medical Center, Part of Maastricht University, 6211 LK Maastricht, The Netherlands; c.reutelingsperger@maastrichtuniversity.nl; 3 The William Harvey Research Institute, Queen Mary University of London, London EC1M 6BQ, UK; m.perretti@qmul.ac.uk

**Keywords:** mucin-1, claudin-1, *zona occludens*, ERK1/2 pathway, angiogenesis, F-actin polymerization, BeWo spheroids, Ishikawa cells

## Abstract

Embryo implantation into the uterine wall is a highly modulated, complex process. We previously demonstrated that Annexin A1 (AnxA1), which is a protein secreted by epithelial and inflammatory cells in the uterine microenvironment, controls embryo implantation in vivo. Here, we decipher the effects of recombinant AnxA1 in this phenomenon by using human trophoblast cell (BeWo) spheroids and uterine epithelial cells (Ishikawa; IK). AnxA1-treated IK cells demonstrated greater levels of spheroid adherence and upregulation of the tight junction molecules claudin-1 and *zona occludens-1*, as well as the glycoprotein mucin-1 (Muc-1). The latter effect of AnxA1 was not mediated through IL-6 secreted from IK cells, a known inducer of Muc-1 expression. Rather, these effects of AnxA1 involved activation of the formyl peptide receptors FPR1 and FPR2, as pharmacological blockade of FPR1 or FPR1/FPR2 abrogated such responses. The downstream actions of AnxA1 were mediated through the ERK1/2 phosphorylation pathway and F-actin polymerization in IK cells, as blockade of ERK1/2 phosphorylation reversed AnxA1-induced Muc-1 and claudin-1 expression. Moreover, FPR2 activation by AnxA1 induced vascular endothelial growth factor (VEGF) secretion by IK cells, and the supernatant of AnxA1-treated IK cells evoked angiogenesis in vitro. In conclusion, these data highlight the role of the AnxA1/FPR1/FPR2 pathway in uterine epithelial control of blastocyst implantation.

## 1. Introduction

The endometrium is a critical tissue for the establishment and maintenance of pregnancy, during which it undergoes extensive physiological changes and demonstrates extraordinary plasticity. Cyclic changes in its tissues enable the endometrium to convert to a receptive state, allowing implantation, attachment, and invasion by the embryo through the epithelium into the underlying stromal compartment [1,2]. Embryo implantation is a highly-organized process that involves a receptive epithelium as well as a competent embryo for attachment, which is finely controlled by soluble and membrane-bound factors such as cytokines, prostaglandins, growth factors, and matrix-degrading enzymes as well as adhesion molecules, and transcription factors [2,3]. In order to achieve successful implantation, crosstalk between a receptive uterus and a competent blastocyst can only occur during a limited time span, known as the “window of implantation” [4,5]. During this short period of time, which occurs approximately 6 to 10 days after ovulation in humans [1,6], the uterus undergoes structural and functional remodeling, mainly via the modulation of estradiol and progesterone. Basically, uterine receptivity is improved when estradiol levels decrease and high levels of progesterone are present [3]. Under these specific conditions, glycoproteins are expressed and highly secreted to prepare the endothelium for embryo attachment, tight junctions are reinforced, and angiogenesis occurs [2,7]. 

Annexin A1 (AnxA1) is a 37 KDa protein that belongs to the calcium and phospholipid-binding protein family within the annexin superfamily. A wide range of cells secretes AnxA1, including those of the innate immune system, as well as epithelial and cancer cells. AnxA1 mediates physiological processes in the body [8,9] although its secretion is highly augmented during challenging processes, such as inflammation and cancer [10,11]. The most well-recognized functions of AnxA1 are its potent anti-inflammatory and pro-resolution activities in the context of the innate immune response, during which glucocorticoids and cytokines induce synthesis and secretion of AnxA1 to halt inflammation and induce its resolution [12,13]. The binding of extracellular AnxA1 to G-protein coupled seven-domain transmembrane formylated peptide receptors (FPRs), especially type 2 (FPR2), is the most described and proven anti-inflammatory mechanism of AnxA1 [14,15,16,17]. Binding of AnxA1 to FPR2 induces rapid heterotrimeric G protein dissociation into the α and βγ subunits. βγ subunit downstream transduction signals, such as those mediated via phospholipase Cγ (PLCγ), result in activation of Ras family proteins and, in turn, activation of the mitogen-activated protein kinase (MAPK) pathway, particularly that of the extracellular signal-regulated kinases (ERK)-1/2. Activation of these later pathways leads to Ca^2+^ mobilization and activation of protein kinase C (PKC) [18,19]. 

Previous data have correlated high levels of AnxA1 in human uterine tissue during gestation and in the seminal fluid [20,21], while lower amounts of AnxA1 have been found in the amnion and placenta at delivery [22,23,24,25,26]. More recently, our group has linked AnxA1 to pregnancy. Specifically, AnxA1-genetically deficient mice (AnxA1^−/−^) presented an increased number of blastocysts and implantation sites, resulting in an increased number of pups delivered [27]. Additionally, the uterine microenvironment of AnxA1^−/−^ mice displayed an inflammatory profile, including a higher content of neutrophils and M1 macrophages as well as enhanced levels of pro-inflammatory cytokines, especially IL-6 [28]. These findings suggest that AnxA1 may play a crucial role in the maintenance of the uterine microenvironment, particularly in relation to maintenance of a receptive environment during implantation [27]. To further understand this process, in the current study we have elucidated the direct actions of AnxA1 on the initial events of blastocyst implantation in cultured human uterine epithelial cells.

## 2. Materials and Methods

### 2.1. Cell Lines 

The human uterine epithelial cell line Ishikawa (IK) was purchased from Banco de Células do Rio de Janeiro. IK cells were maintained in Dulbecco’s Modified Eagle Medium (DMEM; #12100046, Gibco, Carlsbad, CA, USA) supplemented with 10% heat-inactivated fetal bovine serum (FBS; #2024-06, Gibco), 2 mM L-glutamine (#25030081, Gibco) 1 mM pyruvate (#11360-070, Gibco) and 1% antibiotic solution containing streptomycin and penicillin (#15140-122, Gibco). Human umbilical vein endothelial cells (HUVECs) were donated by Dr. Ricardo José Giordano from the Chemical Institute of the University of Sao Paulo. HUVEC cells were maintained in Roswell Park Memorial Institute (RPMI) 1640 medium (#31800089, Gibco) containing 10% FBS and 1% antibiotic solution containing streptomycin and penicillin. BeWo cells were kindly donated by Professor Ana Campa from the Faculty of Pharmaceutic Sciences, University of Sao Paulo, and maintained in DMEM/F12 medium (#12500-062, Gibco) supplemented with 10% FBS and 1% antibiotic solution containing streptomycin and penicillin. All cells were maintained in an atmosphere of 5% CO_2_ at 37 °C and sub-cultured every 3 days by trypsinization, if necessary.

### 2.2. Cell Treatments

Uterine epithelial cells were seeded in 24-well plates (Corning, New York, NY, USA) and cultured for adhesion over 18 h. Once cells had adhered, the medium was replaced and the cells were either pre-incubated with the culture medium (non-treated [NT], i.e., control) or medium supplemented with Boc-2 (1 μM; #SKU 0215276005, MP Biomedicals, Santa Ana, CA, USA), cyclosporine H (1 μM; #AG CN2 0447-M005, Adipo Gen Life Sciences, San Diego, CA, USA) or WRW_4_ (1 μM; #2262, Tocris Bioscience, Bristol, UK) for 30 min. Following the pre-incubation, AnxA1 (1.35 nM; donated by Professor Chris Reutelingsperger from Faculty of Health, Medicine and Life Sciences, Maastricht University) was added to the cell culture, either in the absence or presence of inhibitors, and incubated for a time period according to the specific assay performed. 

For tube formation assay, uterine epithelial cells were washed three times with warm PBS and pre-incubated with FPR inhibitors (30 min) followed by addition of AnxA1, and cultured for 18 h in RPMI 1640 medium supplemented with 1% bovine serum albumin (BSA; #A9418-10G, Sigma-Aldrich). Afterwards, the supernatant was collected in sterile conditions and used to perform the assay.

### 2.3. Cell Viability Assay

Uterine epithelial cells were seeded at 2.5 × 10^4^ cells/well in 24-well plates and incubated in the absence or presence of different concentrations of AnxA1, Boc-2, cyclosporine H, or WRW_4_ over either 24 or 48 h. Following the incubation period, the medium was carefully removed and 300 μL of 3-(4,5-dimethylthiazol-2-yl)-2,5-diphenyltetrazolium bromide (MTT, 0.5 mg/mL; #M5655, Sigma-Aldrich) was added in each well. Cells were maintained at 37 °C for 3 h, after which the supernatant was removed and 200 μL of dimethyl sulfoxide (DMSO; #276855, Sigma-Aldrich) was added into each well and homogenized for 15 min. Absorbance was determined using a spectrophotometer at 575 nm (SpectraMax M Series, Molecular Devices, San Jose, CA, USA). Results were expressed as the percentage of viable cells relative to NT cells (control).

### 2.4. Flow Cytometry

Flow cytometry experiments were performed to characterize the expression levels of AnxA1, FPR1, and FPR2 in uterine epithelial cells, as well as to investigate the role of AnxA1 on CD61, signal transducer and activator of transcription (STAT)1α, nuclear factor (NF)-κB, ERK1/2, homeobox A-10 (HOXA10), progesterone, and estrogen receptor expressions. Briefly, uterine epithelial cells were seeded at 5 × 10^4^ cells/well and treated as mentioned above. Cells were trypsinized (#T1757, Vitrocell, Campinas, SP, BRA), washed twice in PBS containing 1% BSA (collectively referred to as PBS/BSA). To investigate the expression of AnxA1, HOXA10, progesterone, and estrogen receptors, the cells were fixed overnight at 4 °C using FACS lysing solution (#349202, BD Biosciences, San Jose, CA, USA), then washed with PBS containing 1% glycine (#01A1021.01.AG, Synth, Diadema, SP, BRA), permeabilized with Triton-X (0.001%; #T8787, Sigma-Aldrich), washed with PBS/BSA, and incubated with primary anti-human rabbit antibodies to AnxA1 (1:100; #713400, Thermo Fisher, Waltham, MA, USA), HOXA10 (1:100; #720220, Thermo Fisher), the progesterone receptor (1:500; #IM-0558, Rhea Biotech, Campinas, SP, BRA), or the estrogen receptor (1:250; #IM-0557, Rhea Biotech) overnight at 4 °C. Next, cells were washed with PBS/BSA and incubated with secondary goat anti-rabbit antibodies conjugated to Alexa Fluor 488 (1:500; #A11008 Invitrogen) for 40 min in the dark at room temperature (RT). To investigate STAT1α and ERK1/2 expressions, the cells were fixed in cold methanol for 30 min at RT, permeabilized in 0.1% Triton-X for 20 min at RT, and then incubated with primary anti-total STAT1α (1:50; #9172, Cell Signaling, Boston, MA, USA), anti-phospho-STAT1α (1:50; #9167, Cell Signaling), anti-total ERK1/2 (1:200; #ab54230, Abcam, Burlingame, CA, USA) and/or anti-phospho ERK1/2 (1:200; #ab214036, Abcam) antibodies overnight at 4 °C. After this incubation, the cells were blocked with PBS containing 2% FBS, and incubated with secondary anti-rabbit-phycoerythrin (1:200; #ab97070, Abcam) or anti-mouse-fluorescein isothiocyanate (FITC, 1:200; #ab6785, Abcam) goat antibodies for 1 h in the dark at RT. In order to analyze FPR1, FPR2, CD61 and NF-kB expression, the cells were washed twice in PBS/BSA and incubated with specific antibodies as follows: FPR1-PE (1:100; #FAB3744P BD Biosciences, Minneapolis, MN, USA), FPR2-FITC (1:100; #bs3654R FITC; Bioss, Boston, MA, USA), CD61-FITC (1:50; #555753; BD Biosciences) or NF-kB (1:100; #0465R, Biolegend, San Diego, CA, USA) for 40 min in the dark at RT. The cells were then washed and resuspended in PBS at the end of each protocol. Samples were subjected to flow cytometric analysis in a BD Accuri C6 flow cytometer taking 10,000 events into consideration and using CSampler software (BD Pharmingen, CA, USA).

### 2.5. Proliferation Assay

The proliferation assay was performed using a Live/Dead Viability/Cytotoxicity Kit for mammalian cells (#L3224, Thermo Fisher). Briefly, uterine epithelial cells were seeded in 24-well plates at a concentration of 1 × 10^4^ cells/well and incubated in culture medium containing 0.5% BSA for 24 h prior to treatment. Following the incubation, the cells were washed and treated with medium containing either 0.5%, 2%, or 10% BSA in the absence or presence of AnxA1 (1.35 nM) for 24 or 48 h. Next, the cells were washed with PBS, trypsinized, and then incubated using the Live/Dead Viability/Cytotoxicity Kit according to the manufacturer’s instructions. Samples were analyzed using a BD Accuri C6 flow cytometer and 10,000 events were considered in the analysis using CSampler software (BD Pharmingen).

### 2.6. Immunofluorescence

Uterine epithelial cells were seeded at a concentration of 5 × 10^4^ cells/well on glass coverslips inside the wells of 24-well plates and treated as mentioned above. Cells were fixed in cold methanol for 30 min at −20 °C, after which the methanol was removed, and the cells were maintained at −20 °C until the assay was performed. Briefly, cells were washed in PBS and incubated overnight at 4 °C in the presence of anti-Muc-1 (rabbit anti-human; #bs-4763R, Bioss), anti-claudin-1 (rabbit anti-human; #ab15098, Abcam), anti-*zona occludens*-1 (ZO-1; goat anti-human; #PA5-19090, Thermo Fischer), or anti-AnxA1 primary antibodies. Following this incubation, the cells were washed with PBS/BSA and incubated with donkey anti-goat and goat anti-rabbit secondary antibodies conjugated to either Alexa Fluor 568 (#A11011, Thermo Fisher) or FITC (#A11008, Thermo Fisher), respectively. The coverslips containing the cells were removed from the 24-well plates, inverted, and mounted on glass slides in 5 μL of Vectashield (#H-1200, Vectorlabs, Burlingame, CA, USA). The slides were maintained at 4 °C and images were acquired using an Axio Zeiss microscope and analyzed with ImageJ software (NIH, Bethesda, MD, USA).

### 2.7. ELISA

Uterine epithelial cells were seeded at 3–5 × 10^4^ cells/well and treated as mentioned above. The supernatant from these preparations was used to quantify the expression of IL-6 (#555220 BD OptEIA, BD Biosciences Pharmingen), AnxA1 (#MBS704042, MyBiosource, San Diego, CA, USA) and vascular endothelial growth factor (VEGF; #KHG0111, Thermo Fisher) through ELISA, according to the manufacturer’s instructions. Expression level results from the ELISA were expressed in terms of pg/mL.

### 2.8. Trophoblast Spheroid

The method of trophoblast spheroid growth was adapted from a previous study [29]. Briefly, in order to obtain spheroids, 50 μL agarose solution (1.5%; #A9539, Sigma-Aldrich) was added to 96-well plates. After solidifying, BeWo cells were seeded at a concentration of 1 x 10^4^ cells/well and maintained in an atmosphere of 5% CO_2_ at 37 °C for 72 h. Following the incubation, individual spheroids were visualized through optic microscopy. The viability of spheroids was determined using the Live/Dead Viability/Cytotoxicity Kit (Thermo Fisher).

### 2.9. Implantation Assay

The BeWo spheroid implantation model used here was adapted from previous studies [29,30,31]. Briefly, uterine epithelial cells were plated in 96-well plates at a concentration of 2.5 × 10^4^ cells/well and, after adherence, the cells were incubated for 1 h in either the control medium or media containing Boc-2 (1 μM), cyclosporine H (1 μM), or WRW4 (1 μM), and then incubated with AnxA1 (1.35 nM) throughout the implantation time. The spheroids (one spheroid/well) were gently transferred onto adhered uterine epithelial cells and this co-culture was maintained in a humid atmosphere at 5% CO_2_ and 37 °C for 2 h. Following this incubation period, the wells were filled up to the brim with culture medium and the plates were sealed with an adhesive film for microplates, inverted, and then centrifuged at 30× *g* at RT for 5 min. After centrifugation, the plates were kept inverted while they were taken from the centrifuge and examined under a Leica DMi1 inverted microscope (Leica, Shinagawa, Tokyo, Japan) for the presence of the spheroids. The spheroids that disappeared during the centrifugation process were considered to be unattached, and the results were expressed as the percentage of attached spheroids.

### 2.10. Confocal Microscopy

#### 2.10.1. F-Actin Expression

Uterine epithelial cells were seeded at a concentration of 5 × 10^4^ cells/well on a 24-well plate and then treated as mentioned above. Next, the cells were washed in PBS and fixed in 2% paraformaldehyde for 20 min at RT. The cells were then washed in PBS, incubated with rhodamine phalloidin (#R415, Thermo Fisher) for 20 min in the dark at RT, and then washed in PBS. The intensity of fluorescence was detected using high-content imaging with a GE IN Cell Analyzer 2200 (GE Healthcare Life Sciences, Chicago, IL, USA) and quantified with IN Carta^TM^ image analysis software (GE Healthcare Life Sciences).

#### 2.10.2. AnxA1 Expression

AnxA1 expression at the implantation site was evaluated in C57bl/6 mice of 5 to 6 weeks of age. For this purpose, female mice were caged overnight with male mice (3:1) and successful mating was verified the following morning. The presence of a vaginal plug was designated as day 0.5 of gestation. The animals were maintained and bred at the Animal House at the School of Pharmaceutical Sciences, University of Sao Paulo (Brazil). Chow (Quimtia, Colombo, PR, Brazil) and water were made available to the mice *ad libitum*. All animals were housed in a temperature-controlled room (22–25 °C and 70% humidity) with a 12-h light–dark cycle. All procedures were performed according to the Brazilian Society for the Science of Laboratory Animals (SBCAL) and approved by the Institutional Animal Care and Use Committee from the Faculty of Pharmaceutical Sciences of the University of Sao Paulo (Protocol number 557).

For confocal microscopy, the uterus was removed at day 5.5 of gestation following euthanasia of the mice via isoflurane overdose. Samples of the implantation sites were fixed in 4% buffered paraformaldehyde for 72 h at 4 °C, washed in Tris-buffered saline (TBS) and incubated with AnxA1 polyclonal antibody (#713400, Thermo Fisher) or only TBS (negative control) for 24 h. Next, these tissues were incubated with goat anti-rabbit antibodies conjugated with Alexa Fluor 488 (#A11008, Thermo Fisher) and DAPI (10 μg/mL; #D9542, Sigma-Aldrich) for 4 h, at RT in the dark, and then analyzed using a Confocal Zeiss LSM-780-NLO microscope (Carl Zeiss, Jena, Germany).

### 2.11. Tube Formation

The tube formation assay was performed as detailed in previous studies [32,33]. Briefly, HUVEC cells were serum-starved for 24 h in RPMI 1640 medium with 1% BSA. Next, the cells were trypsinized, harvested, and plated on a 96-well plate at a density of 2.5 × 10^4^ cells/well on 100 μL Matrigel coating (#356237, Corning). The cells were incubated for 4 h with uterine epithelial cell-free supernatant that was previously obtained according to item 2.2. HUVEC cells, maintained in RPMI 1640 containing 10% FBS, were used as the positive control (data not shown). Photomicrographs (5X magnification) were obtained using a Leica DMI1 optical microscope (Shinagawa, Tokyo, Japan), and closed units (polygons) were considered in the count. Twelve fields were count per well.

### 2.12. Statistical Analyses

The data were expressed as mean ± standard error of the mean (SEM) and comparisons were made between the experimental groups using a one-way ANOVA followed by either the Tukey test or Bonferroni test for multiple comparisons using GraphPad software version 5. A *p* value < 0.05 was used to denote statistically significant differences.

## 3. Results

### 3.1. Uterine Epithelial Cells Express FPRs 1 and 2 and Secrete AnxA1

To validate our study, we first confirmed that uterine epithelial cells express and secrete AnxA1, and express its receptors, FPR1 and FPR2 (Figure S1). The secretion of AnxA1 was not detected from other epithelial cell lineages, such as Caski and Siha cells, and low levels were detected for HeLa (Figure S1B). Additionally, the concentration-response curves demonstrated that AnxA1, Boc-2, cyclosporine H, and WRW_4_ did not affect the cellular viability under any of the concentrations employed in our studies following either 24 or 48 h of incubation (Figure S2A,C–E). Moreover, AnxA1 did not alter the cellular proliferation (Figure S2B). Using these data, effective concentrations of FPR agonists and antagonists were chosen to proceed with the further investigations, specifically 1 μM of Boc-2, cyclosporine H, and WRW_4_, and 1.35 nM of AnxA1.

### 3.2. AnxA1 Increased the Number of Implanted Trophoblast Spheroids

BeWo spheroids were cultured on uterine epithelial cells in order to mimic embryo implantation in vitro (Figure S3A). Of note, BeWo spheroid viability was confirmed by observation of both a higher number of viable (green; Figure S3B,D) and lower number of dead cells (red; Figure S3C,D).

**Figure 1:**
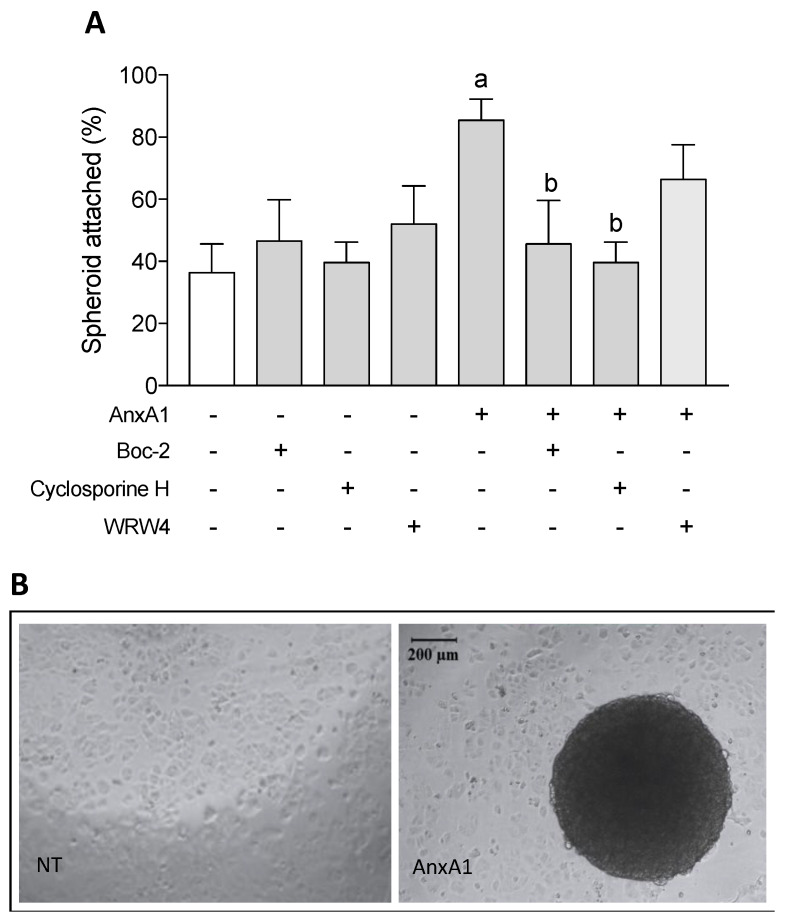
AnxA1 increased BeWo spheroid attachment via FPR1 on uterine epithelial cells. Uterine epithelial cells were treated with FPRs antagonists during 1 h and AnxA1 was added with spheroids. Uterine epithelial and spheroids were co-cultured during 2 h, and the percentage of adhered spheroids were calculated and considered as attached. (−) means absence and (+) means presence of treatments (**A**). Representative image of non-treated (NT) and AnxA1-treated uterine epithelial cells containing or not a spheroid is shown in (**B**). The data are expressed as mean ± standard error of 10 experiments. ^a^ *p* < 0.05 vs. NT; ^b^ *p* < 0.05 vs. AnxA1.

The in vitro implantation assay showed that NT (i.e., control) uterine epithelial cells demonstrated 36.4% spheroid adherence after 2 h of incubation. Similar adherence is observed when cells were treated with Boc-2, cyclosporine H or WRW_4_. AnxA1 treatment evoked a large increase in spheroid adherence, as 85.4% of the spheroids attached to the uterine epithelial cells following the treatment. This effect was reversed when cells were co-incubated with either cyclosporine H or Boc-2 with AnxA1. WRW_4_ did not affect the improved adherence evoked by AnxA1 (Figure 1A). A representative image of the in vitro spheroid adhesion assay is shown in Figure 1B.

### 3.3. AnxA1 Induced Muc-1 Expression in Uterine Epithelial Cells via FPR1 and FPR2

**Figure 2:**
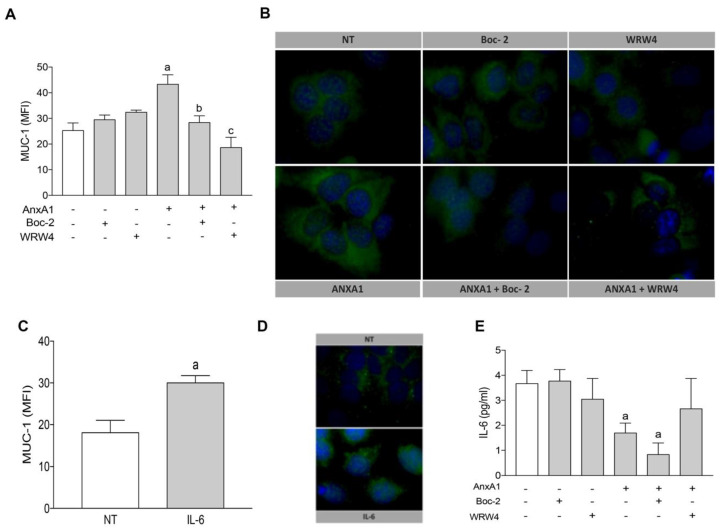
AnxA1 controlled Muc-1 expression on uterine epithelial cells via FPR1/FPR2, independent of IL-6 secretion. Muc-1 expression on uterine epithelial cells was determined 24 h after incubations (**A**,**B**). Muc-1 expression was determined 24 h after IL-6 treatment (**C**). IL-6 secretion was determined in the supernatant of uterine epithelial cells 24 h after treatments (**D**). (−) means absence and (+) means presence of treatments. The data are expressed as mean ± standard error of mean of three to five independent experiments. ^a^ *p* < 0.05 vs. NT; ^b,c^ *p* < 0.05 vs. AnxA1.

Mucins are glycoproteins that line the surfaces of organs exposed to the external environment, including the lung, gut, eyes, and uterus [34]. It has been shown that, in humans, mucin-1 (Muc-1) acts as a scaffold and ligand for selectins present on the blastocyst in order to facilitate attachment [35,36]. The data obtained here show that, in uterine epithelial cells, AnxA1-induced expression of Muc-1 was abrogated by simultaneous incubation with Boc-2 or WRW4 (Figure 2A). Representative images of the immunofluorescence studies are shown in Figure 2B.

Since IL-6 is a key cytokine involved in blastocysts implantation and Muc-1 expression [37,38], we hypothesized that AnxA1 may control Muc-1 expression via IL-6. Indeed, we confirmed IL-6 increased Muc-1 expression by uterine epithelial cells (Figure 2C,D), although IL-6 secretion was impaired in cells treated with AnxA1 (Figure 2E). Taken together, these data show an IL-6-independent mechanism by which AnxA1 impacts on Muc-1 expression, and that such actions are mediated via FPRs.

### 3.4. AnxA1 Induced Claudin-1 and Zona Occludens-1 Expression in Uterine Epithelial Cells via FPR1 and FPR2

**Figure 3:**
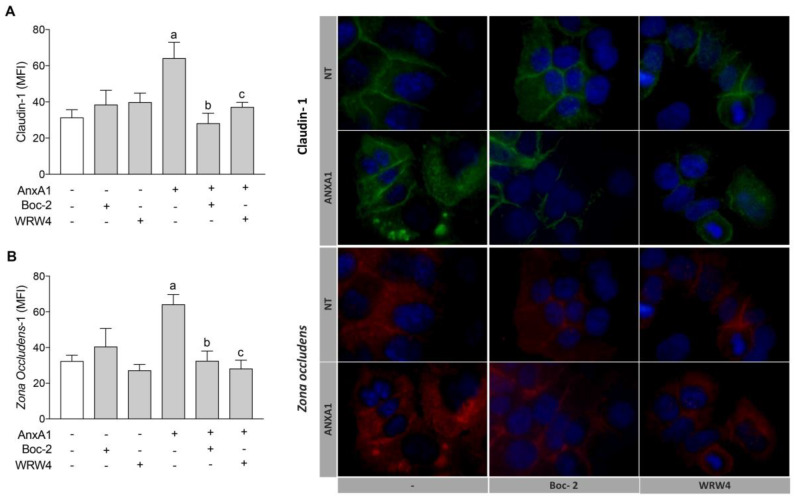
AnxA1 controlled claudin-1 and ZO-1 expressions on uterine epithelial cells via FPR1 and FPR2. Claudin-1 (**A**) and ZO-1 (**B**) expressions on uterine epithelial cells were determined 24 h after incubations. Representative images of claudin-1 and ZO-1 immunofluorescence are shown. (−) means absence and (+) means presence of treatments. The data are expressed as mean ± standard error of mean of three to five independent experiments. ^a^ *p* < 0.05 vs. NT; ^b^ *p* < 0.01 and ^c^ *p* < 0.05 vs. AnxA1.

Claudin-1 is a member of the junctional complex and is associated with cytoplasmic plaque proteins in the *zona occludens* (ZO), which is crucial for maintaining the integrity of the uterine epithelium [7]. AnxA1 treatment increased claudin-1 and ZO-1 expression in uterine epithelial cells, and this effect was abrogated by co-incubation with Boc-2 or WRW_4_ (Figure 3A,B, respectively). Representative images of claudin-1 and ZO-1 immunofluorescence are shown in Figure 3.

### 3.5. Increased Muc-1 and Claudin-1 Expression Evoked by AnxA1 Was Supported by the MAPK Pathway Activation in Uterine Epithelial Cells

**Figure 4:**
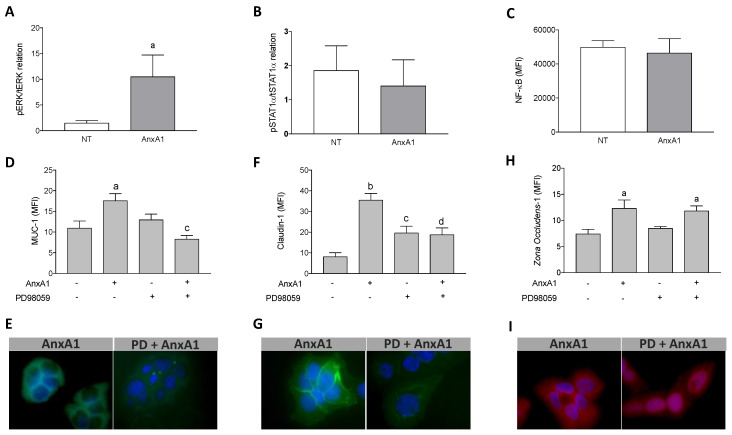
AnxA1 controlled Muc-1 and Claudin-1 expressions on uterine epithelial cells via ERK1/2 phosphorylation. The effect of AnxA1 on ERK (**A**) and STAT1α (**B**) phosphorylation and NF-κB expression (**C**) were investigated by flow cytometry. The inhibition on ERK1/2, evoked by PD98059 incubation, was investigated on Muc-1 (**D**), Claudin-1 (**F**) and ZO-1 (**H**) expressions using immunofluorescence. Representative images of Muc-1, Claudin-1 and ZO-1 are shown in (**E**), (**G**) and (**I**), respectively. (−) means absence and (+) means presence of treatments. The data are expressed as mean ± standard error of mean of three to five independent experiments. ^a^ *p* < 0.05 and ^b^ *p* < 0.01 vs. NT; ^c^ *p* < 0.05 and ^d^ *p* < 0.01 vs. AnxA1.

ERK1/2, STAT1α, and NF-κB are some of the signaling molecules connected to Muc-1, claudin-1, and ZO-1 expression [31,39,40,41]. Furthermore, AnxA1 produces its actions mainly through the MAPK, JAK/STAT, and NF-κB signaling transduction pathways [9,42,43]. We observed that AnxA1 treatment increased ERK1/2 phosphorylation in uterine epithelial cells (Figure 4A) but did not modify STAT1α phosphorylation (Figure 4B) or the p65 subunit of NF-κB (Figure 4C) compared to the control cells (NT). Moreover, our findings showed that the pharmacological blockade of ERK1/2 phosphorylation, by pre-incubation with PD98059, abrogated the increment of Muc-1 and claudin-1 expressions induced by AnxA1 in uterine epithelial cells (Figure 4D,F, respectively). In contrast, pre-incubation of uterine epithelial cells with PD98059 did not block the ZO-1 expression evoked by AnxA1 (Figure 4H). Representative images of Muc-1, claudin-1 and ZO-1 immunofluorescence are shown in Figure 4E,G,I, respectively.

### 3.6. AnxA1 Increased F-Actin Polymerization in Uterine Epithelial Cells via FPR1

**Figure 5:**
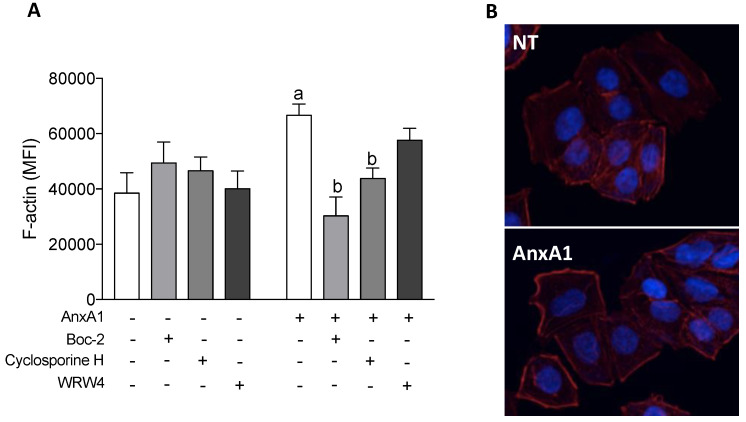
AnxA1 induced F-actin polymerization via FPR1. F-actin was quantified on uterine epithelial cells 2 h after incubations, using phalloidin-rhodamine by confocal microscopy. (−) means absence and (+) means presence of treatments (**A**). Representative images of F-actin polymerization are shown (**B**). The data are expressed as mean ± standard error of mean of three to five independent experiments. ^a^ *p* < 0.05 vs. NT; ^b^ *p* < 0.05 vs. AnxA1.

F-actin is connected to the ZO-1 and is important in stabilizing the tight junctions [44,45]. AnxA1 treatment increased F-actin polymerization in comparison to the control treatments (NT; dotted line), and this effect was inhibited by co-treatment with Boc-2 or cyclosporine H. Co-incubation of cells with WRW_4_ and AnxA1 did not modify F-actin polymerization (Figure 5A). Representative images are shown in Figure 5B.

### 3.7. AnxA1 Controls Endothelial Tube Formation and VEGF Secretion via FPR2

**Figure 6:**
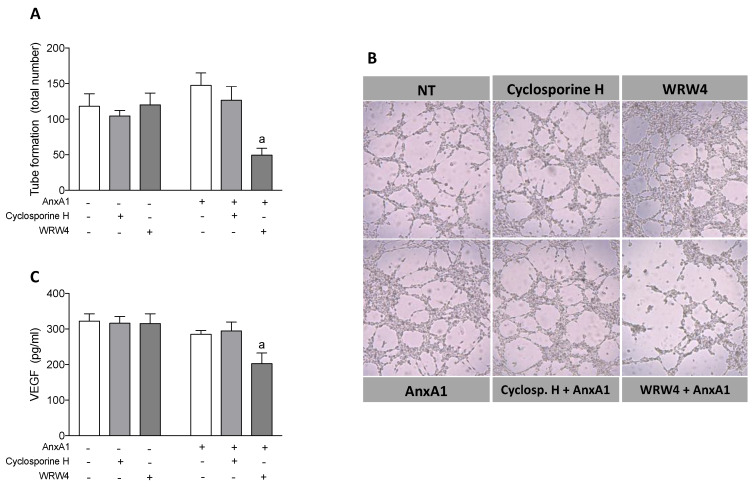
Supernatant of uterine epithelial cells treated with AnxA1 plus WRW_4_ presented reduced levels of VEGF and reduced HUVEC tube formation. Supernatant was obtained from uterine epithelial cells previously treated with cyclosporine H or WRW_4_ in the absence or presence of AnxA1. HUVEC was incubated with supernatants and tube formation was evaluated 4 h later (**A**). Representative images of tube formation are shown in (**B**). The levels of VEGF in the uterine cells supernatant were quantified 4 h after incubations (**C**). (−) means absence and (+) means presence of treatments. The data are expressed as mean ± standard error of mean of at least three to five independent experiments. ^a^ *p* < 0.05 vs. AnxA1.

Angiogenesis is a fundamental step in the implantation process, and thus, required for pregnancy to progress [46]. Therefore, we investigated the role of substances secreted by uterine epithelial cells after treatment with AnxA1 and/or FPR inhibitors on tube formation of HUVECs, referred to as in vitro angiogenesis. Cyclosporine H or WRW_4_ did not alter the number of HUVEC tubes *per se*. The supernatant of uterine epithelial cells, which had previously been incubated with AnxA1 or AnxA1 plus the FPR1 antagonist cyclosporine H, did not modify the formation of tubes by HUVECs. In contrast, the supernatant of uterine epithelial cells treated with AnxA1 and WRW_4_ did markedly reduce the number of tubes formed (Figure 6A). Representative images demonstrating this are depicted in Figure 6B.

VEGF is a fundamental growth factor in angiogenesis. Therefore, to understand the mechanisms linked to the altered tube formation observed, we quantified the levels of VEGF in the supernatant previously obtained from uterine epithelial cells incubated with AnxA1 and FPR blockers. In accordance with the results obtained from the HUVEC tube formation experiments, VEGF levels were not altered by cyclosporine H, WRW_4_, AnxA1, or co-incubation with cyclosporine H and AnxA1. In contrast, significantly lower levels of VEGF were quantified in the supernatants from uterine epithelial cells treated with both WRW_4_ and AnxA1 (Figure 6C).

### 3.8. AnxA1 Is Physiologically Expressed on Uterine Epithelial Cells and the Blastocyst during In vivo Implantation

**Figure 7:**
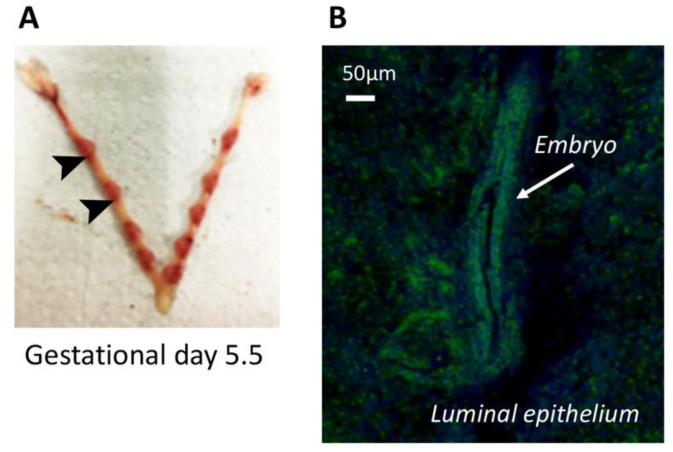
AnxA1 is expressed at embryo-implantation site in vivo. Implantations sites were obtained from C57bl/6 at gestational day 5.5 (**A**). Representative image of AnxA1 expression at implantation site (arrow) and luminal tissue by confocal microscopy is shown (**B**).

To corroborate our results on the functional involvement of AnxA1 in uterine receptivity and embryo implantation, the expression of AnxA1 was monitored by confocal microscopy of implantation sites obtained from C57bl/6 mice on gestational day 5.5 (Figure 7A). The data obtained showed that AnxA1 was broadly expressed by both the luminal epithelium and the embryo (Figure 7B). A representative image of the negative control is shown in Figure S5**.**

## 4. Discussion

Failure of the blastocyst to implant in the uterine wall is a putative cause of unsuccessful pregnancy. Although large volumes of data have been published regarding this process, research thus far has failed to produce an effective treatment that supports the uterus’ receptivity to implantation. Blastocyst apposition and attachment to the uterus involve the actions of a diverse range of molecules and intracellular signaling processes that are not unique in themselves, but play unique roles in each step of the process [2]. Moreover, ethical concerns limit the ability for human studies, and thus, our current understanding of human embryo–endometrium interactions is limited, although experimental models and in vitro cell systems have been designed and are currently being used to help understand the complexity of this phenomenon [29,30]. About 30% of human pregnancies end in miscarriages, the vast majority of which occur in the early phase of gestation [47], thus emphasizing the crucial importance of further studies in this field. Here, we highlight the role that the AnxA1 protein plays, via activation of G-protein coupled receptors (specifically FPRs), in preparing the uterine epithelium for blastocyst implantation using an in vitro model. We found that, indeed, AnxA1 application favors blastocyst attachment by inducing the expression of proteins by the uterine epithelium that control the paracellular flux, structure, and adhesiveness of the uterine wall, as well as promoting angiogenesis.

Progesterone binds to nuclear receptors in the cells of the uterine epithelium and stroma, and activates the transcription factor HOXA10 to induce cell proliferation in the uterine wall, a process which is required for implantation of the blastocyst [48,49]. It has been shown the HOXA10 gene promoter contains a progesterone-responsive element, implying that HOXA10 is a direct target of the progesterone receptor [50,51]. Moreover, activation of the transcription factor HOXA10 induces the expression of the adhesion molecule CD61, a pivotal beta-3 integrin for blastocyst attachment in the epithelium [52]. In humans, high plasma progesterone levels have been associated with increased levels of CD61 in uterine biopsy samples, suggesting a role for CD61 as a biomarker of uterine receptivity [53]. A role for AnxA1 in the fundamental progesterone/HOXA10/CD61 pathway of blastocyst implantation was initially disregarded after studies demonstrated that AnxA1 treatment failed to induce intracellular expression of the progesterone receptor or HOXA10 (Figure S4), epithelial proliferation (Figure S2) or expression of CD61 (Figure S4).

Conversely, our data show that AnxA1, via activation of FPRs, induces expression of Muc-1. Muc-1 is a glycoprotein that is highly expressed in the receptive human endometrium and, subsequently, is removed from the apical epithelium at the location of implantation, while continuing to be expressed by cells neighboring the implantation site [2,54,55,56]. Moreover, abnormal Muc-1 expression has been detected in women suffering from recurrent miscarriages or fertility problems [57]. Muc-1 expression is controlled by various molecules, such as IL-6 [37,38] and high levels of progesterone, either alone or in combination with estradiol in vivo and in vitro [58,59]. Here, we assumed that other pathways are involved in AnxA1-induced Muc-1 expression, as AnxA1 reduced secretion of IL-6 by uterine epithelial cells while no alterations of progesterone and estrogen receptors were observed in this cell line. Data regarding the action(s) of AnxA1 on mucin expression are not currently available, and the results presented here may help further investigations concerning the effects of AnxA1 on Muc-1 expression in other epithelial cells, since Muc-1 is known to be present on the surface of most epithelial cells and to be involved in processes such as microbe invasion, inflammation, and fibrosis [60,61].

Similar to the results observed for Muc-1 expression, we detected increase in claudin-1 and ZO-1 expression following treatment with AnxA1, and these effects were blocked by co-treatment with FPR1 and FPR2 antagonists. Claudin-1 is a transmembrane adhesion protein that binds to cytoplasmatic adapter proteins of the ZO-1 and forms tight junctions [62,63,64]. Crucial roles for both proteins have been described in regulating transepithelial permeability to small molecules and ions [63,65], as well as cell growth and differentiation under various conditions [42,66,67,68,69]. Recently, claudin-1 and ZO-1 have been revealed to be key components involved in uterine receptivity, as tight junctions become reinforced during blastocyst implantation [7,70]. Here, we show that the AnxA1/FPR1/2 pathway contributes to strengthening claudin-1 and ZO-1 between uterine epithelial cells, suggesting a role by AnxA1 in controlling paracellular flow. Accordingly, the reinforcement of tight junctions contributes to the retention of uterine secretions within the lumen of the uterus, which is important for nourishing the embryo and favoring implantation [7,70,71].

The downstream effects of AnxA1 on intracellular pathways in uterine epithelial cells involve MAPK signaling, but not engagement of the transcription factors STAT1α or NF-κB. Increased ERK1/2 phosphorylation was detected following AnxA1 treatment, while inhibition of ERK1/2 phosphorylation reverted the changes in expression of Muc-1 and claudin-1 induced by this AnxA1 treatment. In fact, it has been shown that both Muc-1 and claudin-1 are pre-transcriptionally induced via the MAPK/ERK pathway [72,73,74]. Furthermore, MAPK signaling is a common pathway implicated in various actions induced by AnxA1 [9,13,75,76]. However, AnxA1 controls ZO-1 expression independently of ERK1/2 phosphorylation, potentially via improving F-actin polymerization as interactions between ZO-1 and the actin cytoskeleton have been reported in epithelial cells [44,77]. The pivotal role of AnxA1 on cytoskeletal reorganization of epithelial cells has been demonstrated under physiological conditions, and a deficiency of AnxA1 expression with consequent destabilization of focal adhesions and tight junctions has been implicated as a possible mechanism of various diseases [78,79,80,81,82,83].

Angiogenesis is crucial for successful implantation and placentation, and is critical during decidualization [46]. Angiogenesis begins early in the course of implantation and is supported by pro-angiogenic molecules [84]. The role of AnxA1 in angiogenesis has been shown in different models of cancer [85,86]. Moreover, the N-terminal AnxA1 peptide Ac_2-26_ has been demonstrated to increase endothelial tube formation by increasing proliferation, migration, and actin polymerization in a manner similar to that induced by VEGF-A [83]. Our data show AnxA1, via FPR2, induces the secretion of angiogenesis factors by uterine epithelial cells, resulting in HUVEC tube formation. In our model, VEGF was identified as one of these angiogenic factors as its concentrations were reduced if uterine epithelial cells were treated with FPR2 antagonist in association with AnxA1. This is in line with recent work by Ferraro and co-workers [87], who demonstrated a functional link between AnxA1 and reparative macrophage phenotype in settings of heart failure, consequent to VEGF release from the immune cell. Therefore, we conclude that AnxA1, via FPR2, induced epithelial secretion of VEGF which, subsequently, modulated angiogenesis in the endothelium, demonstrating a complementary mechanism by which AnxA1 is able to support embryo implantation.

The direct action of AnxA1 on uterus epithelium demonstrated here seems to sound controversial to the high number of pups delivered by AnxA1 knockout mice [27]. In addition to the differences found in epithelium of mice and humans, we infer the exacerbated inflammation detected in the uterus microenvironment in AnxA1-deficient mice may favor the implantation process. It is known that elevated levels of pro-inflammatory cytokines, such as IL-6, favor the implantation of blastocysts [37]. Therefore, we infer AnxA1 is pivotal in the implantation phase, by controlling the inflammation that maintains the microenvironment to support a compatible blastocyst implantation, and also, by inducing the required signaling in the epithelium to trigger the adhesive properties. 

In conclusion, this study unveils the existence of an intricate mechanism by which AnxA1 controls embryo implantation through regulating expressions and functions of key molecules linked to uterine receptivity, integrity, and angiogenesis. AnxA1 interacts with FPRs to activate members of MAP kinases and modulate the epithelial cytoskeleton, resulting in a uterine environment conducive for embryo implantation in the epithelium. Moreover, AnxA1 is also connected to the dynamic interplay between the uterine epithelium and endothelium, crucial for embryo implantation, posterior decidualization, and consequently, successful pregnancy.

## Supplementary Materials

The following are available online at https://www.mdpi.com/2073-4409/9/5/1188/s1, Figure S1: AnxA1 expression and secretion and FPR1 and FPR2 expressions by uterine epithelial cell lineage Ishikawa. Figure S2: AnxA1, Boc-2, cyclosporine H and WRW4 did not modify uterine epithelial cells viability. Figure S3: BeWo spheroid viability. Figure S4: AnxA1 did not modify progesterone and estrogen receptors, HOXA10, and CD61 expressions. Figure S5: Representative image of negative control of embryo implantation site.
