# Supplementary material for: Annexin A1/Formyl Peptide Receptor Pathway Controls Uterine Receptivity to the Blastocyst
Source: Cells. 2020 May 11;9(5):1188. doi: 10.3390/cells9051188 (PMC7291299; doi:10.3390/cells9051188)
Supplement: Supplementary file 1 [file cells-09-01188-s001.pdf]

Article

Annexin A1/Formyl Peptide Receptor Pathway Controls Uterine Receptivity to the Blastocyst

Cristina B. Hebeda [1], Silvana Sandri [1], Cláudia M. Benis [1], Marina de Paula-Silva [1], Rodrigo A. Loiola [1], Chris Reutelingsperger [2], Mauro Perretti [3] and Sandra H. P. Farsky [1,*]

[1] Department of Clinical and Toxicological Analyses, School of Pharmaceutical Sciences, University of Sao Paulo, São Paulo, Brazil; crisbh@gmail.com (C.B.H.); ssandris@gmail.com (S.S.); claudiabenis@usp.br (C.M.B.); mpsilva.bio@gmail.com (M.d.P.-S.); rodrigoazl@gmail.com (R.A.L.); sfarsky@usp.br (S.H.P.F.)

[2] Faculty of Health, Medicine and Life Sciences, Part of Maastricht University Medical Center, Part of Maastricht University, Maastricht, Netherlands; c.reutelingsperger@maastrichtuniversity.nl

[3] The William Harvey Research Institute, Queen Mary University of London, London, United Kingdom; m.perretti@qmul.ac.uk

* Correspondence: sfarsky@usp.br; Tel.: +55-(11)-3091-2197

Legend for Supplementary Figures

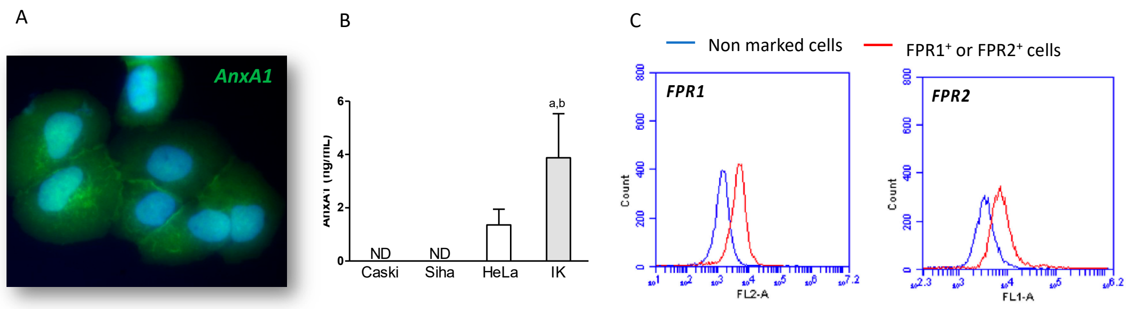

Figure S1. AnxA1 expression and secretion and FPR1 and FPR2 expressions by uterine epithelial cell lineage Ishikawa. AnxA1 expression was determined by immunofluorescence (A). Secretion of AnxA1 by Caski, Siha, HeLa and Ishikawa cells was determined by ELISA (B). FPR1 and FPR2 (C) expressions were determined by flow cytometry. All data are represented as mean ± standard error of the mean (SEM) of 3 independent experiments. $^{a,b}p<0,05$ vs. Caski and Siha.

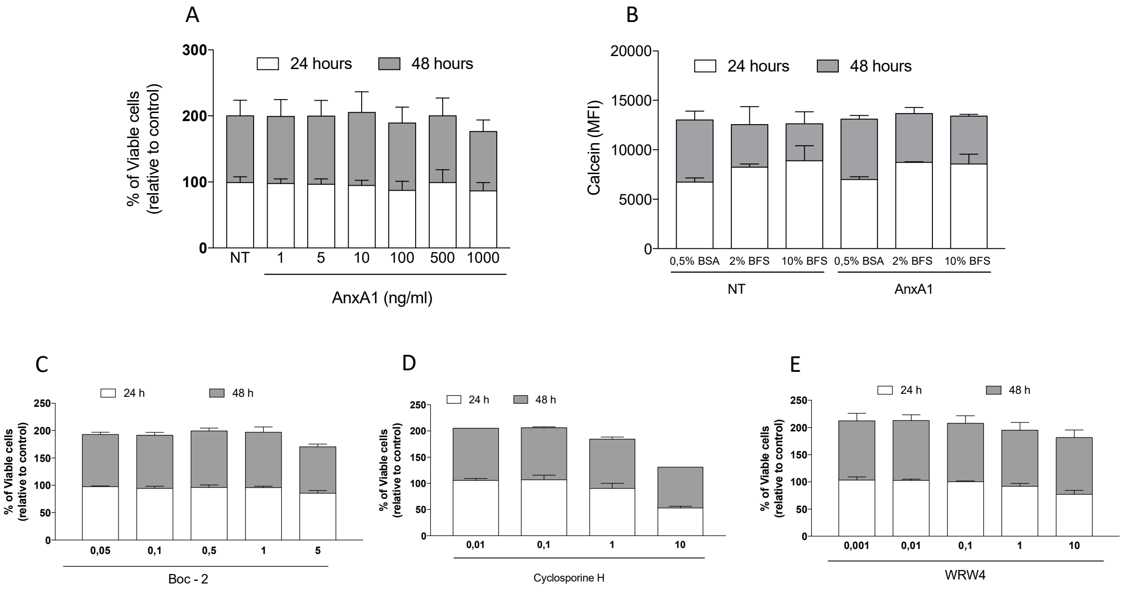

Figure S2. AnxA1, Boc-2, cyclosporine H and WRW4 did not modify uterine epithelial cells viability. Viability of uterine epithelial cells was quantified by MTT assay 24 or 48 h after AnxA1 (A), Boc-2 (C), Cyclosporine H (D) and WRW4 (E) treatments. AnxA1 did not modify cell cycle at different conditions (B). All data are represented as mean ± standard error of the mean (SEM) of 3 independent experiments.

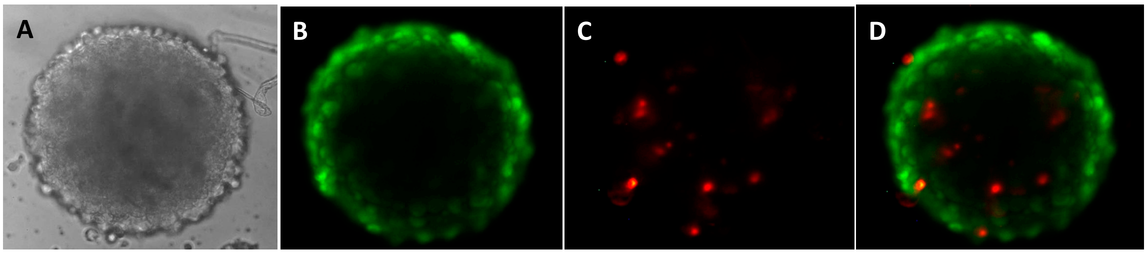

Figure S3. BeWo spheroid viability. Cell Trace was used to determine cell viability and (A) represents an spheroid visualized at inverted microscopy, (B) represents viable cells (green), (C) represents death cells (red) and D show a merged image.

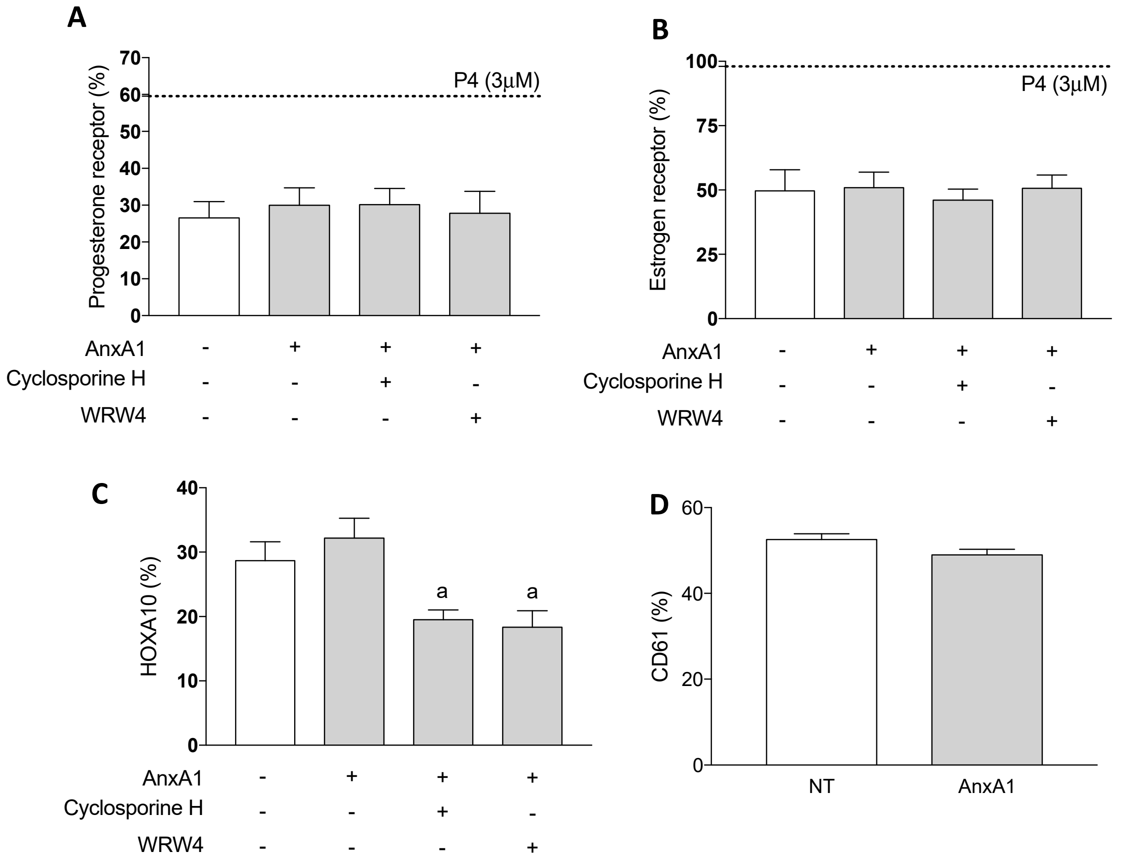

Figure S4. AnxA1 did not modify progesterone and estrogen receptors, HOXA10, and CD61 expressions. Progesterone (A) and estrogen receptor (B), HOXA10 protein (C) and CD61 expressions (D) were quantified by flow cytometry. Uterine epithelial cells were treated with cyclosporine H and WRW4 in absence or presence of AnxA1 (1.35 nM). As positive control of estrogen and progesterone receptors expression, cells were treated with progesterone (P4; 100 ng/mL; dotted line). The data are expressed as mean ± standard error of mean of at least three to five independent experiments.

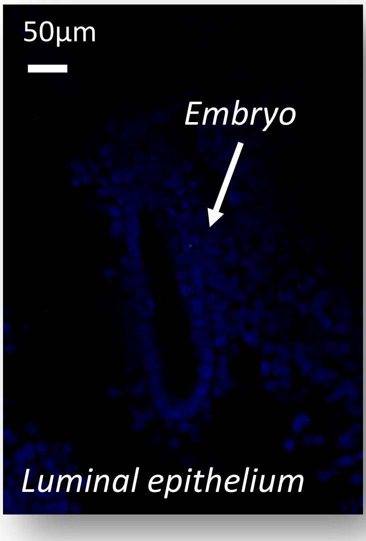

Figure S5. Representative image of negative control of embryo implantation site. Tissue was incubated with TBS instead primary antibody and then with secondary goat anti-rabbit antibody conjugated with Alexa Fluor 488 and DAPI. Implantation sites were analyzed using a Confocal Zeiss LSM-780-NLO microscope (Carl Zeiss, Jena, Germany).
